# New insights on single-neuron selectivity in the era of population-level approaches

**DOI:** 10.3389/fnint.2022.929052

**Published:** 2022-09-28

**Authors:** Francesco Edoardo Vaccari, Stefano Diomedi, Matteo Filippini, Kostas Hadjidimitrakis, Patrizia Fattori

**Affiliations:** ^1^Department of Biomedical and Neuromotor Sciences, University of Bologna, Bologna, Italy; ^2^Alma Mater Research Institute for Human-Centered Artificial Intelligence, University of Bologna, Bologna, Italy

**Keywords:** mixed selectivity, posterior parietal cortex, neural networks, motor control, neural code, multisensory integration

## Abstract

In the past, neuroscience was focused on individual neurons seen as the functional units of the nervous system, but this approach fell short over time to account for new experimental evidence, especially for what concerns associative and motor cortices. For this reason and thanks to great technological advances, a part of modern research has shifted the focus from the responses of single neurons to the activity of neural ensembles, now considered the real functional units of the system. However, on a microscale, individual neurons remain the computational components of these networks, thus the study of population dynamics cannot prescind from studying also individual neurons which represent their natural substrate. In this new framework, ideas such as the capability of single cells to encode a specific stimulus (neural selectivity) may become obsolete and need to be profoundly revised. One step in this direction was made by introducing the concept of “mixed selectivity,” the capacity of single cells to integrate multiple variables in a flexible way, allowing individual neurons to participate in different networks. In this review, we outline the most important features of mixed selectivity and we also present recent works demonstrating its presence in the associative areas of the posterior parietal cortex. Finally, in discussing these findings, we present some open questions that could be addressed by future studies.

## Introduction

Historically, the “neuron doctrine” stated that the single neurons are not only the structural but also the functional units of the nervous system. Tightly related to this view is the concept of the receptive field defined as “a specific feature of the sensory world that activates it [each neuron] and defines its function” (Yuste, 2015). The search for a strictly quantifiable receptive field and optimal stimulus for each neuron led to a huge expansion of knowledge about the functional organization of many brain regions and the discovery of the columnar organization in several of them that culminated with the emblematic “ice cube” model in the early visual cortex (Hubel and Wiesel, 1962). A direct consequence of this view was the idea that a neural population was necessarily divisible into sub-populations or cell categories composed of cells specialized (selective) to code for a different variable.

However, over the years, this approach could not fully explain the neural activity of many associative and motor cortices, and discrepancies started to emerge. For example, the fact that motor neurons are tuned by either “high-level” (trajectory, speed, etc.) and “low-level” (muscle force, join torques, etc.) movement features or the non-stationarity of the correlations between single-cell activity and motor output parameters, represented strong challenges for the traditional selectivity perspective (Scott, 2008; Omrani et al., 2017). Thanks to technological advances that expanded the possibility to record from multiple neurons simultaneously, a new view shifted the attention from the single neurons to the neural populations (Yuste, 2015; Kalaska, 2019). This new approach has made significant advances so much so that the motor cortical areas are now viewed as “dynamical systems” that exhibit low-dimensional, rhythmic activity optimized to generate the proper behavior, disregarding correlations between single cell activity and movement parameters (Churchland et al., 2012; Shenoy et al., 2013; Michaels et al., 2016; Omrani et al., 2017; Gallego et al., 2018; Kalaska, 2019). It is evident that the “traditional” selectivity principle needs to be revised considering new evidence and conceptual advances.

## The mixed selectivity

**Figure 1:**
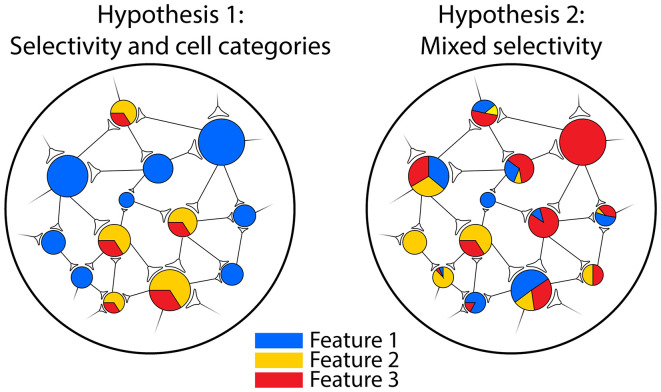
Schematic representation of neural populations with different types of encoding. Left: a population composed of neurons selective for one feature of the stimulus (blue circles) or responding to a fixed combination of features (yellow/red circles). In this situation, two cell categories can be identified. Right: a population characterized by mixed selectivity for the three features: a few units are strictly selective for a specific feature (e.g., the wide red circle), but most of them respond to different combinations of features. In this case, it is not possible to identify cell categories and the information is distributed across the network.

The concept of “mixed selectivity” has been put forward over the last 10 years to reconcile old ideas with recent evidence in a unique framework. This term, coined for the first time to describe the activity of the prefrontal cortex (PFC) neurons in a complex cognitive task, was defined as the neuron's capacity to encode a combination of internal and external variables (Rigotti et al., 2013). Notably, in this perspective, how the information is encoded by individual neurons matters (Fusi et al., 2016; Parthasarathy et al., 2017; Johnston et al., 2020). In fact, it has been shown that the integration of multiple variables is carried out in a non-linear fashion from a mathematical point of view and therefore the abbreviation NMS (Non-linear Mixed Selectivity) is often used. On the contrary, a “linear mixed selectivity” would not guarantee the advantageous properties of NMS (see below) and it has been found only marginally in neurophysiological data (Dang et al., 2022). For simplicity, hereafter, we use the term “mixed selectivity” to intend the NMS. The mixed selectivity implies the mixing of features with “relative weights” that vary from cell to cell, resulting functionally in cell-categories free neural populations and the distributed encoding of information within the network (Raposo et al., 2014; Blanchard et al., 2018). Accordingly, it assumes the presence of a continuous spectrum of modulations from which the emergence of a neuron highly selective only for one feature is rare, but still a possible epiphenomenon (e.g., the “Jennifer Aniston neuron,” Quiroga et al., 2005; for a graphical scheme, see Figure 1).

### Reasons to multiplex information

It has been shown that the nonlinear integration of multiple inputs is the optimal way to either *encode* information about a feature or *decode* it downstream (Rigotti et al., 2013; Fusi et al., 2016).

First, the information encoding recalls the concept of “neural representation” according to which salient and measurable features of the external environment are directly correlated with the neurons' activity (Vilarroya, 2017). Neural representations based on a non-linear mapping between stimulus and firing rate can prevent the response saturation that occurs when a neuron reaches its maximum physiological limit and is not able to transmit further information. This is not guaranteed with linear encoding. Moreover, the interaction between multiple features in the framework of mixed selectivity could produce different responses to the same stimulus in different contexts (Rigotti et al., 2013). In this regard, for example, parietal and prefrontal neurons were recently found to exhibit spatial tuning for the location of a visual stimulus depending on the context (i.e., as a cue or the match stimulus; Dang et al., 2022). Finally, a mixed code provides a better tradeoff of information channeled vs. energy cost, requiring more neurons, but the same number of spikes to be transmitted with respect to a “pure,” selective code (Johnston et al., 2020).

Second, the information encoded in the firing activity must be decoded by the downstream structures for further computations. The mixed selectivity expands the processing capabilities of the network by creating more heterogeneous activity patterns. Indeed, the dimensionality of a neural representation does not necessarily coincide with the mere number of neurons, but rather with the intrinsic complexity of the data structure. It has been shown that, given a constant number of units, the neural representations produced by NMS neurons are much more complex (high-dimensional) and they can support many more readouts rather than populations of linear selective cells (Fusi et al., 2016). Even if a linear encoding scheme can still carry relevant information for a specific context, its low-dimensional neural representation of the input prevents a simple readout in other contexts. Moreover, the latter encoding type would require precise, hard-wired connections between units, whereas a circuit with distributed information (such as in the case of NMS) allows the estimation of the relevant parameters by any arbitrary group of units (Ganguli and Sompolinsky, 2012; Raposo et al., 2014). Another advantage of mixed selectivity is the reliability of the neural code which is less affected by noise resulting in much fewer decoding errors than a “pure” selective coding (Johnston et al., 2020).

### Mixed selectivity in posterior parietal cortex

The numerous advantages in encoding and decoding explain the fact that the mixed selectivity seems now widely present in the cortex, encompassing sensory (Walker et al., 2011; Rentzeperis et al., 2014; Finkelstein et al., 2018), prefrontal (Rigotti et al., 2013; Fusi et al., 2016; Parthasarathy et al., 2017), and motor areas (Churchland and Shenoy, 2007; Hatsopoulos et al., 2007). Nevertheless, only a few studies directly addressed this issue in other associative cortical regions and the posterior parietal cortex (PPC) in particular.

For simplicity, the several PPC functions can be grouped into three domains (Krumin et al., 2018). The first one regard visuomotor coordination during eye and limb movements (Andersen and Mountcastle, 1983; Andersen and Buneo, 2002; Filimon, 2010; Caminiti et al., 2015; Pisella et al., 2017; Galletti and Fattori, 2018; Hadjidimitrakis et al., 2019). Second, the PPC is involved in decision-making, especially when the choice is guided by vision (Andersen and Cui, 2009; Erlich et al., 2015; Latimer et al., 2015; Goard et al., 2016; Katz et al., 2016; Licata et al., 2017; Krumin et al., 2018). Finally, this cortical region plays an important role in spatial navigation (Nitz, 2006, 2012; Save and Poucet, 2009; Whitlock et al., 2012; Wilber et al., 2014). In this regard, most of the navigation studies have been conducted in rodents, but reports about primates can also be found (Sato et al., 2006; Vass and Epstein, 2013). Given the richness of the information processed by PPC, it is reasonable to assume that its areas present mixed selectivity rather than having subpopulations (i.e., classes or categories) of highly selective neurons. Furthermore, the idea that parietal neurons are non-linearly modulated by multiple stimuli is not completely new. For example, the old concept of “gain field,” a multiplicative interaction between visual receptive field response and eye position, can be thought of in this perspective (Andersen et al., 1985). However, many past works considered the conjunctive tuning for only very few features, and they often did not explicitly try to model the heterogeneity of more complex neural responses, picking as representative the rare “pure” selective units for which something similar to a “receptive field” could be identified.

Here, we present studies that directly addressed the issue of mixed selectivity in PPC in various animal species to provide a brief overview of the ongoing research on this topic.

**Figure 2:**
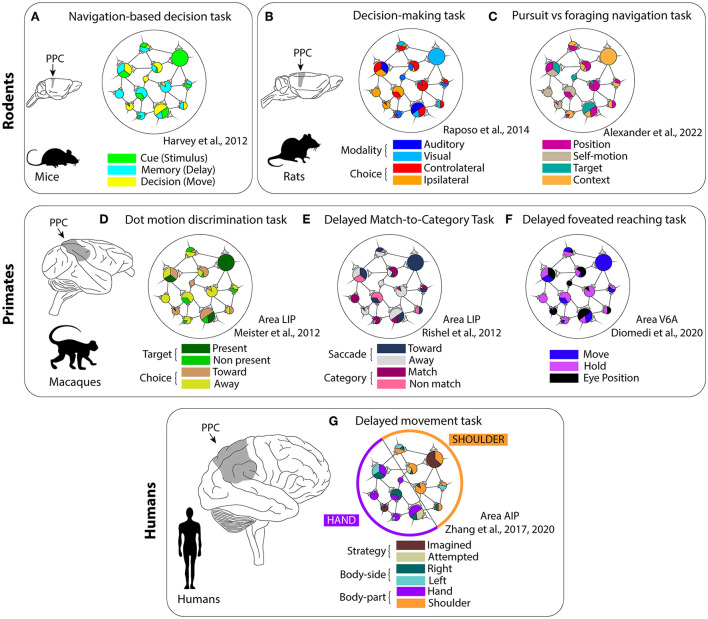
Mixed selectivity studies in the posterior parietal cortex across different animal species. **(A–C)** In rodents; **(D–F)** in macaques; and **(G)** in humans. Due to the heterogeneity of this region, the features tested in each work depended on the parietal area of interest. Mixed selectivity has been found in all studies, but not in the human AIP (“partially mixed selectivity”). **(A)** Navigation-based decision task. **(B)** Decision-making task. **(C)** Pursuit vs. foraging navigation task. **(D)** Dot motion discrimination task. **(E)** Delayed match-to category task. **(F)** Delayed foveated reaching task. **(G)** Delayed movement task.

Several studies reported evidence consistent with the mixed selectivity framework, both at the single-neuron (encoding of multiple information) and at the population level (lack of cell categories, high-dimensional responses) in rodents. For example, Harvey et al. (2012) investigated whether PPC activity could be described in terms of cell categories, or by a more complex structure in mice during a navigation-based decision task. They did not encounter cell classes with homogeneous activity patterns, but, on the contrary, the information about the cue, delay period, and movement choice was carried by the population dynamics through sequential activation patterns (Figure 2A). Similarly, Raposo et al. (2014) recorded from rat PPC during a multisensory decision-making task (Figure 2B). The animals had to discriminate the frequency of stimuli that could be either visual (a series of flashes), auditory (a series of “clicks”) or combined visuo-auditory and respond by selecting between two ports. Neurons only rarely exhibited pure choice or pure sensory modality selectivity, and, in most cases, they showed mixed responses to the task features. However, the population responses were clearly structured and they could be easily decoded by a linear classifier. Recently, individual PPC neurons in rats were found to encode different combinations of spatial position, self-motion, and egocentric target position during a navigation task that included pursuit and free foraging phases (Alexander et al., 2022; Figure 2C). Moreover, neural responses were found to be modulated nonlinearly by context and able to adapt to different distributions of encoded features.

Regarding primates, the macaque PPC has been extensively studied, given the highly similar manual dexterity and brain morphology of this species to humans (Goldring and Krubitzer, 2020). Within PPC, the lateral intraparietal area (LIP) exhibits functions related to saccades planning and execution, visual stimuli processing, decision-making, and evidence accumulation (Andersen and Cui, 2009; Premereur et al., 2011; de Lafuente et al., 2015). Interestingly, Meister et al. (2013) trained macaques to perform a motion direction-discrimination task and make a saccade in the direction cued by the dot motion (relevant information) toward a target that could be visible within the receptive field or not (irrelevant information). The authors reported that single neurons mixed both relevant and irrelevant information (Figure 2D). Moreover, the neural correlates of evidence accumulation were evident only in the population activity as mixed selectivity predicts. In another work, LIP neurons showed modulations both for non-spatial and spatial features (“abstract” categories and saccade targets; Rishel et al., 2013; Figure 2E). Here, an independent encoding for the different features was found in the firing rate of individual cells (categories and saccades), in line with the distributed information expected in the context of the mixed selectivity.

Similarly to LIP, a multitude of different inputs converges to the medial parietal area V6A. V6A is particularly involved in arm-reaching movements both during the planning and execution phases (Hadjidimitrakis et al., 2014; Fattori et al., 2017; Diomedi et al., 2021). Recently, we found that, during foveated delayed reaching movements, spatio-temporal information about the target and task phase was distributed across the network and the population did not cluster in well-defined categories of selective units, according to the mixed selectivity scheme (Diomedi et al., 2020; Vaccari et al., 2021; see also Figure 2F).

Finally, also in the human PPC, it was reported the presence of mixed selectivity (Zhang et al., 2017, 2020; Figure 2G). The authors recorded the neural activity with a multielectrode array implanted in the anterior intraparietal area (AIP) of a tetraplegic patient. The patient was instructed to perform imagined or attempted movements (cognitive strategy) with left or right (body side) different body parts (hand or shoulder). The authors found a particular structure in the combined encoding of the various task variables that they termed “partially mixed selectivity:” different body parts were represented by different subpopulations of cells, but within these subpopulations, the strategy and body side information was randomly distributed. Notably, the mixed representations were preserved by passing from an open-loop experiment to a close-loop control task involving a brain-machine interface (Zhang et al., 2020).

## Discussion

Nowadays, there is increasing interest in the characterization of the role of neural ensembles leveraging the possibility to record large datasets, the extensive use of analytical tools to extract population dynamics (such as dimensionality reduction techniques), and the capacity of artificial neural networks to mimic many features of the real brain (Cunningham and Yu, 2014; Kalaska, 2019). Within this framework dominated by population approaches, the mixed selectivity concept represents an important conceptual advance, since it can reconcile the traditional idea of single neurons' “neural selectivity” with their functioning as units embedded in populations with dynamic interactions. Recent evidence suggests that while this property is widespread in the cortex, its computational benefits are well-grounded in theoretical work (see “Reasons to multiplex information” paragraph).

Since many areas of the posterior parietal cortex exhibit mixed selectivity (see “Mixed selectivity in posterior parietal cortex” paragraph), it is tempting to speculate that the entire PPC is characterized by such computations. However, some results partially disagree (Zhang et al., 2017), suggesting caution in generalizing. Regarding PPC motor functions, further studies should assess if the body part segregation that characterizes the human AIP (Zhang et al., 2017) can be found also in non-human primate AIP and other parietal areas. In this regard, it has been proposed that PPC is organized in intention maps/matching movements regions (Kaas, 2012; Andersen et al., 2014), resembling the organization proposed for motor cortices (Graziano and Aflalo, 2007). If it were the case, the mixed selectivity could be accordingly limited, with specific areas showing NMS only within a type of action, but not responding to other actions. However, in overt contrast to such clear segregation of actions in the PPC, several studies reported the presence of grasping-related signals in areas traditionally associated with reaching (Fattori et al., 2009, 2010; Galletti and Fattori, 2018), as well as reaching-related signals in areas traditionally associated with grasping (Lehmann and Scherberger, 2013). Medendorp and Heed (2019) tried to conceptualize these ideas in a unique framework proposing the existence in PPC of a medio-lateral gradient dominated by action classes but involving tightly interconnected networks and often the same units. These neurons may encode information with a mixed selective mechanism, being able to participate in the various networks in a flexible, context-dependent way.

The complexity of the problem (whether and how mixed selectivity is implemented in PPC) increases when considering the involvement of PPC in all the functional domains that we already mentioned (motor control, decision-making, and navigation). Notably, besides motor control, navigation can also be included in Medendorp and Heed (2019); similarly, all three functions are considered by Kravitz et al. (2011). From these organizing schemes, new hypotheses can be derived regarding which variables are encoded (and eventually mixed) by the neurons of different PPC areas. For example, an open question is whether primate and rodent systems have undergone a differentiation of functions, with primates more specialized in moving eyes, head, and limbs and rodents more specialized in tracking the path traveled and orienting the body in the environment, or whether the disparity in the literature (motor control predominant in primate studies, navigation in rodents) could have produced biased results (Wilber et al., 2014). Supporting the idea of functional differentiation, neurons in rat PPC encode the posture of the entire animal body (Mimica et al., 2018), but similar findings have not been reported yet for primate PPC. Since these aspects have a direct impact on the encoding of single neurons, they should be addressed in future studies.

Very recent reports are highlighting dissimilarities regarding the mixed selectivity of prefrontal and parietal cortices. Zhou et al. (2021) found that PFC neurons exhibited a higher NMS with respect to parietal neurons during a sequential match/non-match task. Similarly, a working memory task modulated a significantly lower amount of NMS units in PPC with respect to the prefrontal cortex (Dang et al., 2022). The authors suggest that these differences could be grounded in anatomical dissimilarities: PFC pyramidal neurons are endowed with the most extended dendritic trees of any other cortical neuron, thus they could represent a perfect substrate to implement NMS. In addition, some task variables (e.g., reward) were less affected by NMS than others both in PFC and PPC, suggesting an influence of the hierarchical rank of the features on their encoding type (Dang et al., 2022).

Finally, future studies that aim to characterize the encoding scheme of parietal neurons should take into consideration two important aspects. First, it is possible that a neuron apparently selective for a single feature during a task (erroneously classified as “selective”) can show responsiveness for other features in different contexts. In other words, at least some selective units could only consist of insufficiently tested mixed neurons, thus task complexity should be adequate to maximize NMS neural modulations. Second, the terms “feature”/“variable” themselves, although they are useful to define neural selectivity, can be quite vague and hide spurious correlations with multiple parameters. In addition, we might be biased to define as selective a neuron that encodes a complex variable which can be decomposed into multiple variables with a similar “semantic meaning” (i.e., cartesian position and its x-y components) and, conversely, to define as mixed a neuron that encodes a complex variable which, however, can be decomposed into multiple variables with a different “semantic meaning” (i.e., cartesian position and context). It is beyond the scope of this review to deal with these issues in detail, but in the future, defining these concepts in formal terms will be necessary to make research on these topics more homogeneous and consistent.

## Author contributions

FV wrote the manuscript draft. SD prepared the figures. PF provided the funding and facilities. All authors edited the manuscript, contributed to the article, and approved the submitted version.

## Funding

This work was supported by Ministero dell'Università e della Ricerca (Italy, PRIN2017-2017KZNZLN) and H2020-EIC-FETPROACT-2019-951910-MAIA.

## Conflict of interest

The authors declare that the research was conducted in the absence of any commercial or financial relationships that could be construed as a potential conflict of interest.

## Publisher's note

All claims expressed in this article are solely those of the authors and do not necessarily represent those of their affiliated organizations, or those of the publisher, the editors and the reviewers. Any product that may be evaluated in this article, or claim that may be made by its manufacturer, is not guaranteed or endorsed by the publisher.
